# Laryngeal Chondrosarcoma as a Rare Cause of Subglottic Stenosis

**DOI:** 10.1155/2014/730643

**Published:** 2014-08-13

**Authors:** Kerem Kökoğlu, Özlem Canöz, Serap Doğan, Emrah Gülmez, İmdat Yüce, Sedat Çağlı

**Affiliations:** ^1^Department of Otolaryngology, Faculty of Medicine, Erciyes University, 38039 Kayseri, Turkey; ^2^Department of Pathology, Faculty of Medicine, Erciyes University, 38039 Kayseri, Turkey; ^3^Department of Radiology, Faculty of Medicine, Erciyes University, 38039 Kayseri, Turkey

## Abstract

Laryngeal chondrosarcoma (CS) is a very rare entity. It is usually seen in 50–80-year olds. It is developed from cricoid cartilage largely. Patients have laryngeal CS complaint of respiratuvar distress, dysphonia, and dysphagia generally. A submucous mass is usually seen in physical examination with an intact mucosa. Distant metastasis is rare in CSs. Main treatment is surgical excision. An 82-year-old patient who has respiratuvar distress is presented in this paper and laryngeal CS is reviewed in the light of the literature.

## 1. Introduction

Laryngeal CS is a rare entity and it is less than 1% of all larynx cancers [1]. Its etiology is unknown but more accepted theory is disorganized ossification of laryngeal cartilages. Radiotherapy to head-neck area and Teflon injection are also implicated as etiology. It is most frequently seen in the range of 50–80 years [2]. The youngest and eldest patients who have laryngeal CS are 33 and 91 years old, in the literature [3]. 75% of laryngeal CS develops from the cricoid cartilage, 17% from thyroid, and % 5 from epiglottis, arytenoid, and the others [1, 4]. Clinical signs vary according to location and size of the tumor. Distant metastasis of CS is late but it is prone to relapse [5].

The aim of this paper is to present a laryngeal CS case and review its diagnosis and treatment in the light of the literature.

## 2. Case Presentation

**Figure 1:**
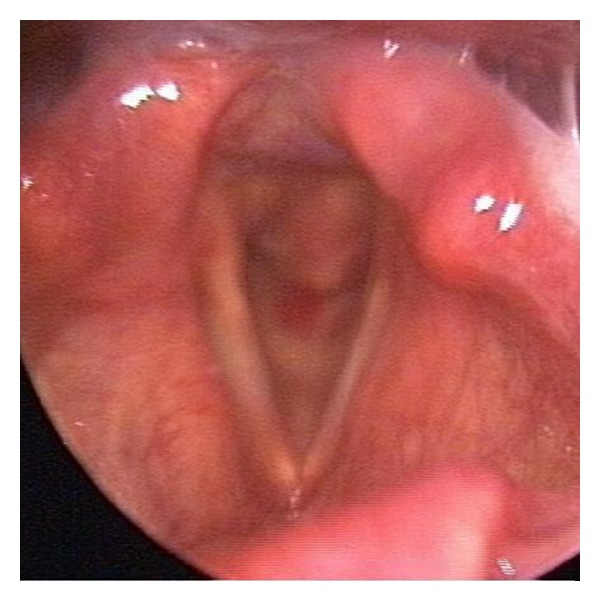
A mass is seen in subglottic space especially the posterior part with an intact mucosa.

**Figure 2:**
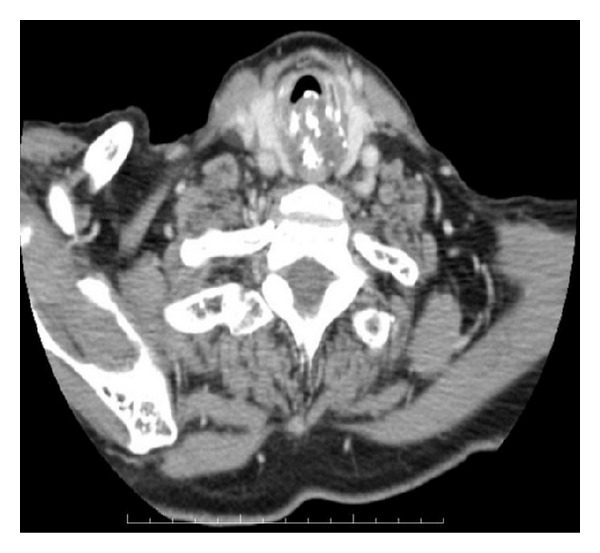
CT imaging. A mass that has popcorn calcification is seen in the posterior part of laryngeal airway.

An 82-year-old patient was admitted to our clinic with a complaint of shortness of breath. She had for 2 years this symptom and it had been worsened. She also had hoarseness and difficulty in swallowing. In her medical history, she was being treated for hypertension and asthma. She was not a smoker or an alcoholic. There was no malignant disease in her relatives. She had stridor and supraclavicular-suprasternal retractions. Her subglottic area was very narrow in physical examination. There was a mass but mucosa on mass was steady. Her laryngeal airway was narrowed by mass in form of expansion (Figure 1). There was no lymphadenopathy in her neck. Tracheotomy was applied urgently and deep biopsy was done from the mass. There was a mass developed from cricoid cartilage in her computed tomography (CT). It had narrowed subglottic region and extended the first tracheal ring. The mass involved hyperdense, characteristic calcified areas like popcorn and contrasted moderately (Figure 2). Biopsy result was grade one CS. Total laryngectomy was performed for treatment. There was no complication in the followup.

## 3. Discussion

Laryngeal CS is most frequently seen in the range of 50–80 years. Male to female ratio is 4/1. Its etiology is still unknown but the most acceptable theory is irregular ossification of laryngeal cartilages. Radiotherapy to head-neck area and Teflon injection can also be a reason [2]. The presented patient was an 82-year-old lady and had no history like irradiation and Teflon injection.

75% of laryngeal CS develops from the cricoid cartilage like the presented patient and 17% from the thyroid cartilage [4]. Clinical findings vary according to location and size of the tumor. Patients may present with shortness of breath, difficulty in swallowing, hoarseness, and neck mass complaints. Duration of symptoms ranges from several weeks to several years [5]. Tumors developed from the cricoid cartilage can cause dysphagia pressing the esophagus [3]. In order to develop dyspnea, laryngeal airway must be narrowing at least 75% [6]. Symptoms can be confused with chronic laryngitis and asthma symptoms [7]. The presented patient was being treated for asthma for 2 years. She had also difficulty in swallowing because of cricoid tumor.

**Figure 3:**
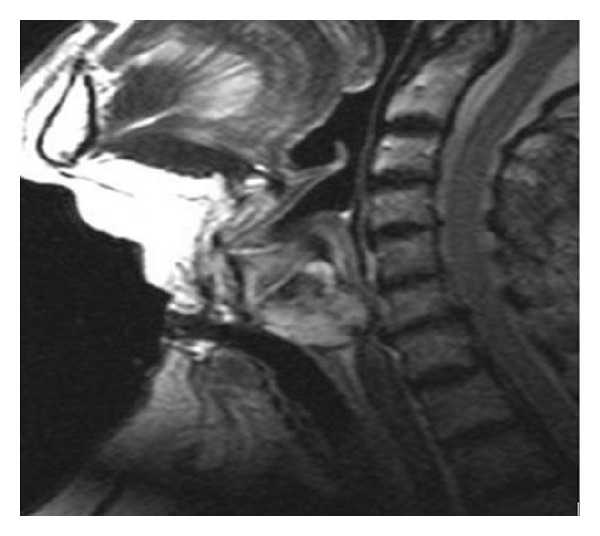
T2 weighted turbo spin-echo sagittal image shows a lobulated, high signal intensity mass indicating chondroid matrix.

A mass covering intact laryngeal mucosa is seen in endoscopic examination. But in advanced cases mucosal ulceration can also be seen [8]. In CT, laryngeal CS is seen as a lobular, hypodense, submucous mass developed from cartilage, including ring-like, stippled, and popcorn calcification, and narrows laryngeal airway. It is isointense according to muscle in T1 scans and hyperintense in T2 scans in magnetic resonance imaging (MRI) [9]. The presented patient had a lobular mass developed from cricoid cartilage and including typical popcorn calcification in CT. In MRI, the mass was hypointense in T1, hyperintense in T2, and warmly holding after intravenous contrast application (Figure 3).

There is no reliable evidence to differentiate between benign chordoma and low-grade CS radiologically [10]. Both masses are developed from cartilage and have similar radiological signs. But lymph node metastases and local invasion could be seen in high-grade CSs. Tracheopathia osteochondroplastica, relapsing polychondritis, laryngeal nodular chondrometaplasia, and other sarcomas like osteosarcoma, synovial cell sarcoma, fibrosarcoma, and malignant fibrous histiocytoma should be considered in the differential diagnosis [11].

**Figure 4:**
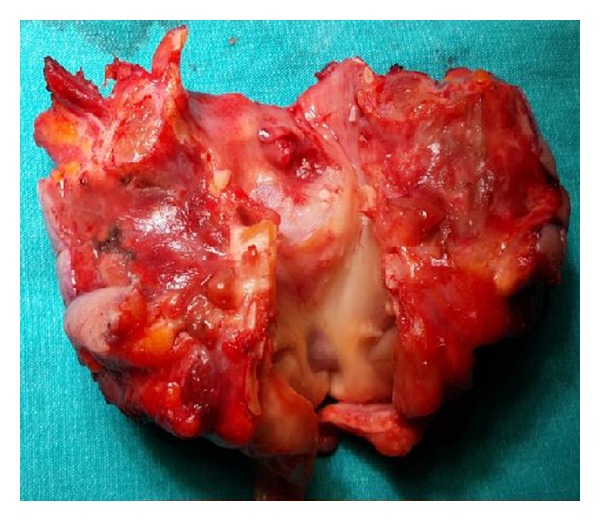
A specimen of total laryngectomy. Larynx was cut from the front and the mass is seen in subglottic area.

**Figure 5:**
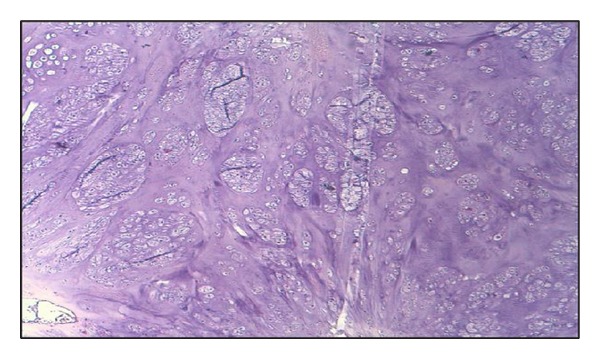
Histopathological image. There is a little more cellularity than usual.

**Figure 6:**
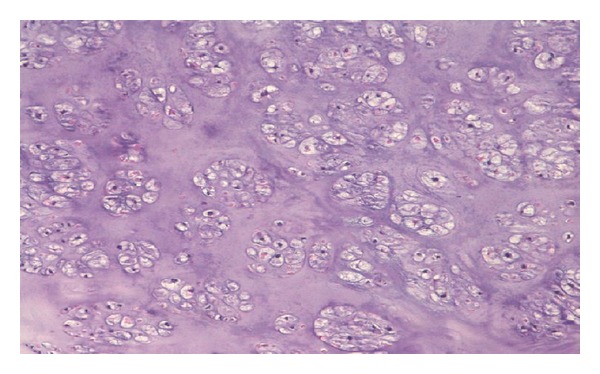
Another histopathologic image in larger magnification. Binuclear cells are seen in places.

For the definitive diagnosis, direct laryngoscopy and biopsy must be done, because submucosal, deep biopsy should be performed [8]. Differentiating between benign chordoma and low-grade CS can also be very difficult histopathologically. The recurrence of a laryngeal cartilaginous tumor is a significant criterion for the diagnosis of malignancy. Laryngeal CSs may be divided into 3 groups: low, intermediate, and high grade. This grading is based on nuclear and cellular atypia, cellularity, and mitosis. Low-grade CS has only a minimally increased cellularity and nuclear atypia in many areas. When it is frequent, binucleation may suggest malignancy [3]. Our patient had a 3 × 2 × 2 cm laryngeal low-grade CS (Figure 4). Binuclear cell number was 34 in 20 large magnification areas. Considering cellularity, 80 cells were seen in every magnification area (Figures 5 and 6).

The main treatment of laryngeal cartilaginous tumors is surgical excision. Surgery that protects laryngeal functions is recommended if possible [1]. But 75% of laryngeal CS develops from cricoid cartilage which is considered crucial for normal laryngeal function and to protect laryngeal functions is not possible mostly [12]. Total laryngectomy is recommended for CS destructing more than half of the cricoid cartilage [13]. Total cricoidectomy protecting laryngeal functions is also defined by de Vincentiis et al. [14]. They mentioned 3 patients who underwent total cricoidectomy with an end-to-end anastomosis between the remaining larynx and the trachea for the treatment of low-grade CS. One of the patients could be decannulated. No recurrence was detected. As a result, total cricoidectomy may be better choice for phonatory and swallowing functions compared to total laryngectomy.

Primary radiotherapy is not recommended for the treatment. A study involving 12 patients who had primary radiotherapy for laryngeal CS showed long-term remission in only two of the patients [15]. Laryngeal CS does not respond to chemotherapy so it is also not recommended as treatment choice [12].

About 2–10% of the reported cases are described as metastatic disease. Lung, bone, and liver metastases have been reported [12]. Mortality due to laryngeal chondrosarcoma is uncommon. Thompson and Gannon showed 4,5% of the patients as a direct result of laryngeal CS [1].

## 4. Conclusion

Laryngeal CS is a very rare and slow-growing entity. Its distant metastasis is also rare. Its symptoms can be confused with asthma and laryngitis. So we must be careful for the differential diagnosis especially in elderly patients.
